# Assessment of Skin Physiology Changes, Efficacy, and Safety of a New Complex Herbal Cream: A 4‐Week Multi‐Center, Open‐Label, Prospective Study of Patients With Sensitive Skin and Rosacea

**DOI:** 10.1111/jocd.71001

**Published:** 2026-07-07

**Authors:** Xiaohong Shu, Yuling Chen, Qiujing Chen, Zhaoxia Li, Wei Huo, Lin Zou, Li Li, Xi Wang

**Affiliations:** ^1^ Cosmetics Evaluation Center West China Hospital, Sichuan University Chengdu Sichuan China; ^2^ Department of Dermatology West China Hospital, Sichuan University Chengdu Sichuan China

**Keywords:** complex herbal cream, erythema, inflammation, rosacea, sensitive skin, skin barrier function, TEWL

## Abstract

**Background:**

Products formulated with *Prinsepia utilis* oil and *Portulaca oleracea* extract have the potential to enhance stratum corneum hydration, decrease transepidermal water loss (TEWL), and alleviate erythema in individuals with sensitive skin.

**Aims:**

To evaluate the efficacy and safety of a newly developed herbal cream in improving signs and symptoms in patients with sensitive skin and rosacea.

**Methods:**

A 4‐week multicenter study was conducted among 104 participants across 5 centers. Participants were classified into the sensitive skin or rosacea group. Parameters were evaluated at 0, 7, 14, and 28 days. Transepidermal water loss rate (TEWL), hydration, pH, sebum, erythema index, clinician erythema assessment, self‐assessment, and dermatology life quality index were evaluated.

**Results:**

At the 28‐day follow‐up, the sensitive‐skin group showed significant improvements compared to the baseline: the erythema index decreased by 8.06% (from 321.73 to 295.80; *p* < 0.001), TEWL decreased by 6.02% (from 31.21 to 29.33 g·h^−1^·m^−2^; *p* < 0.05), and the mean dermatology life quality index score improved from 4.9 ± 4.4 to 2.3 ± 3.0 (*p* < 0.05). Similarly, the rosacea group demonstrated significant improvements: the erythema index decreased by 10.25% (from 356.87 to 320.3; *p* < 0.01), TEWL decreased by 16.21% (from 29.05 to 24.34 g·h^−1^·m^−2^; *p* < 0.01), and the mean dermatology life quality index improved from 5.6 ± 4.1 to 3.1 ± 3.5 (*p* < 0.05).

**Conclusions:**

The newly developed cream demonstrated efficacy in alleviating the signs and symptoms of rosacea and sensitive skin, as evidenced by enhancements in skin barrier function and reductions in inflammation.

**Abbreviations**
CEA: Clinician Erythema Assessment
DLQI: Dermatology Life Quality Index
SC: stratum corneum
TEWL: transepidermal water loss rate
TNF‐α: tumor necrosis factor‐α

## 1 Introduction

Sensitive skin, characterized by sensory responses such as stinging, burning, or itching to normally non‐irritating stimuli, with or without erythema [1, 2], shares key features with rosacea, a chronic inflammatory dermatosis, including neurovascular dysfunction, inflammation [3, 4], and impaired barrier function, which increases transepidermal water loss (TEWL) and irritant susceptibility [5, 6, 7, 8, 9]. Effective management must address both barrier repair and inflammation.

Standard treatments can induce irritation, with frequent symptom recurrence. Thus, barrier‐supporting, soothing skincare may serve both as primary therapy for sensitive skin and an adjunct in rosacea. In this study, we evaluated the efficacy and safety of a novel cream formulated with a unique combination of herbal actives (Prinsepia utilis oil, *Portulaca oleracea* extract, *Physalis alkekengi* extract) and exogenous ceramides to improve skin barrier function and reduce inflammation. Unlike conventional barrier repair products, this formulation integrates synergistic mechanisms: Prinsepia utilis oil and *Portulaca oleracea* extract enhance barrier parameters and reduce erythema [10], while exogenous ceramides support lipid barrier repair [11]. Additionally, *Physalis alkekengi* extract provides specific anti‐inflammatory effects [12, 13]. This combination offers a multi‐targeted approach to support barrier integrity and alleviate inflammation, assessed in individuals with sensitive skin and rosacea.

## 2 Materials and Methods

### 2.1 Study Design

A 4‐week, multicenter, open‐label, prospective study was conducted at five centers. This study was approved by the appropriate ethics review committee (2021年审(360)号), and all participants provided written informed consent. The study protocol was registered at ClinicalTrials.gov (ChiCTR2300071789).

### 2.2 Test Substances

The complex herbal cream used in this study is a commercially available product manufactured by Beitaini Bio‐technological Co. Ltd. (Kunming, China). The cream was supplied directly by the manufacturer as a finished product and used according to their instructions. Its key active ingredients include *Portulaca oleracea* extract (3%), *Physalis alkekengi* calyx extract (0.25%), and a combination of exogenous ceramides, Prinsepia utilis oil, and cholesterol formulated at a 3:1:1 ratio.

### 2.3 Participants

Adult participants (16–65 years) were classified into two groups: sensitive skin and rosacea. Sensitive skin was confirmed using the lactic acid sting test (LAST) [14]. At room temperature (21°C ± 1°C), 50 μL of 10% lactic acid solution was applied to the nasolabial fold and one cheek (randomly selected). Participants reported their sensory response (stinging sensation) at 2.5 and 5.0 min post‐application using a four‐point scale (0, no; 1, mild; 2, moderate; 3, severe stinging). A total score (sum of 2.5‐min and 5‐min scores) ≥ 3 indicated positivity for sensitive skin. The LAST is a well‐established and common diagnostic tool for selecting participants with sensitive skin in dermatological research, particularly for evaluating the irritant or soothing potential of topical formulations [2]. The LAST offers greater objectivity than relying solely on subjective self‐reporting while still including a self‐reported sensory component [1, 15]. Moreover, the LAST has high sensitivity and specificity, with approximately 90% of individuals who self‐identify as having sensitive skin also testing positive in the LAST [15]. Patients diagnosed with mild or moderate rosacea were included in the rosacea group. As each center recruited 24 patients (12 per group), 120 patients were planned across the five centers. Clinical drugs approved for patients with rosacea are tetracycline, hydroxychloroquine, compound glycyrrhizin, and antihistamines. Participants received one or more of these approved medications according to their individual clinical indications; the type and dose of medication were not standardized but were documented during the study. Participants were provided with the study product for self‐application. Participants began using the product before the clinical treatment period at least twice a day until the end of the test period. No other products with similar activities were allowed alongside the study product. Sunscreen use was standardized: participants with habitual sunscreen use continued their original product (brand and SPF/PA value documented), whereas those without such habits used physical sun protection (e.g., umbrella). Compliance was monitored by daily records, follow‐up visits, and product weighing.

### 2.4 Evaluation

Participants were followed up at baseline and on days 7 ± 2, 14 ± 2, and 28 ± 2. We evaluated the efficacy of the cream based on biophysical skin measurements, participants' self‐assessment, and physicians' assessment. All skin measurements were conducted in a climate‐controlled room at a constant temperature of 21°C ± 1°C and relative humidity of 40%–60%. Participants were instructed to cleanse their face using a standardized protocol before resting in the climate‐controlled room for at least 30 min to allow for skin acclimatization to the ambient conditions. This procedure was followed at each follow‐up visit to minimize environmental and physiological variability.

**FIGURE 1:**
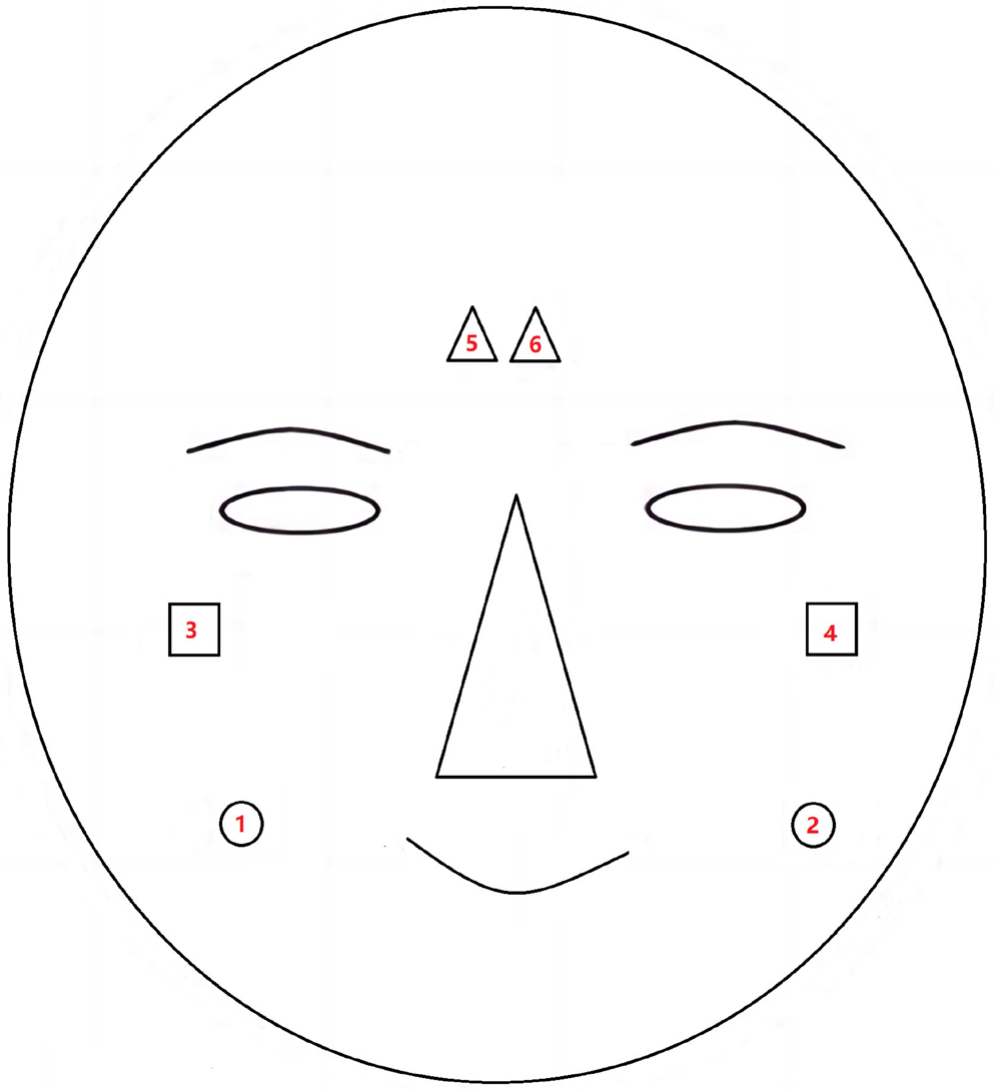
Test sites for skin biophysical measurements. Hydration of the stratum corneum: 3, 4; transepidermal water loss (TEWL): 3, 4; pH: 1, 2; sebum secretion: 5, 6; and erythema index: 3, 4.

The biophysical skin measurements included facial photography, stratum corneum (SC) hydration, TEWL, pH, sebum secretion, and erythema index (EI). SC hydration was measured using a Corneometer (CM825, Courage and Khazaka, Cologne, Germany), which assesses hydration through capacitance, exploiting the high dielectric constant of water. As both sensitive skin and rosacea often involve barrier disruption and reduced hydration, this provides a reliable measure of skin moisture dynamics. TEWL was measured using a Tewameter (TM300, Courage and Khazaka), which quantifies the rate of water evaporation (g·h^−1^·m^−2^) via an open‐chamber method. Elevated TEWL reflects compromised barrier function, which is common in these skin conditions, making it a widely accepted standard for non‐invasively assessing barrier integrity [16]. Skin surface pH was measured using a skin pH meter (pH 900, Courage and Khazaka), employing a planar glass electrode for potentiometric measurement. Maintaining an acidic surface pH (4.0–6.0) is crucial for optimal barrier function, and the elevated pH levels (6.0) often observed in patients with rosacea and sensitive skin correlate with barrier impairment [17, 18]. Sebum secretion was measured using a Sebumeter (SM815; Courage and Khazaka), which quantifies skin surface lipids (μg/cm^2^) photometrically via sebum‐absorbing tape. Alterations in sebum levels affect the skin barrier [19]. The EI was measured using a narrow‐spectrum reflectance spectrophotometer (Mexameter MX18, Courage and Khazaka), which measures hemoglobin absorption via spectral analysis at specific wavelengths (568, 660, and 880 nm), providing an objective measure of vasodilation and inflammation [20]. Together, these instruments provide objective, reproducible, and non‐invasive assessments of key skin parameters for monitoring sensitive skin and rosacea and evaluating therapeutic interventions. Each measurement was performed in triplicate, and the mean was used for analysis. The sides of the test face are shown in Figure 1.

The front, left, and right sides of the face were photographed using the VISIA system (Canfield Scientific Inc., Fairfield, NJ, USA). Each photograph was captured using three light sources.

Two physicians performed a clinician erythema assessment (CEA) to assess the severity of facial erythema before and after using the product.

Questionnaires were provided to evaluate skin symptoms, such as prickling sensation, burning sensation, itching, pain, self‐perceived dryness, and tightness, graded as 0 (none), 1 (light), 2 (medium), and 3 (heavy). At the first and last follow‐up, the Dermatology Life Quality Index (DLQI) was used to measure quality of life. The DLQI consists of 10 questions for self‐evaluation of life experiences in the past week, including symptoms and feelings, daily activities, leisure, work and school, personal relationships, and treatment. Each item was rated on a 4‐point scale (0–3), with a total score of 0–30 points [21]. A higher score indicates a greater impact on quality of life [21]. Additionally, tolerance and adverse reactions were assessed throughout the follow‐up period.

### 2.5 Statistical Analysis

Analyses were performed using SAS version 9.4 (SAS Institute Inc., Cary, NC, USA). Data were managed in SAS. Continuous variables are presented as mean ± standard deviation (SD), and categorical variables as frequency (percentage). All statistical tests were two‐sided with a significance level of α = 0.05. Given the exploratory nature of this study, no formal correction for multiple comparisons was applied; therefore, all reported *p*‐values are nominal and the findings should be interpreted as hypothesis‐generating rather than confirmatory. Where applicable, effect sizes with corresponding 95% confidence intervals (CIs) are reported to facilitate interpretation beyond null hypothesis testing.

## 3 Results

### 3.1 Patients

**FIGURE 2:**
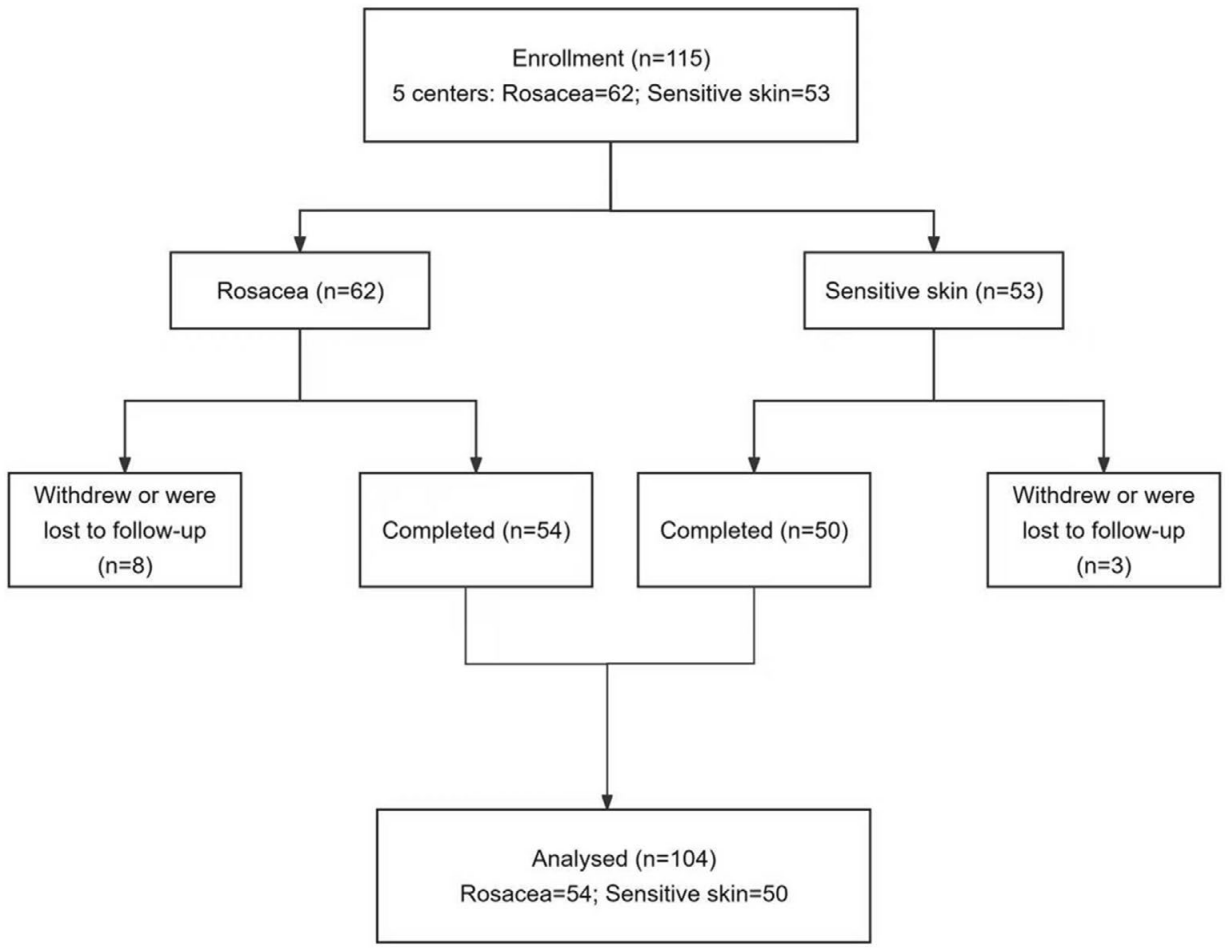
CONSORT flow diagram showing participant progression through the study. Of 115 enrolled participants (53 with rosacea, 62 with sensitive skin), 11 discontinued (3 and 8 per group, respectively), resulting in 104 participants included in the final analysis.

A total of 115 patients (62 with rosacea and 53 with sensitive skin) were enrolled at the five centers, among whom 11 did not complete the trial for various reasons. Thus, 104 participants completed the trial (54 with rosacea and 50 with sensitive skin) (Figure 2). Participant demographic characteristics, TEWL, SC hydration, pH, EI, and CEA scores at baseline are presented in Table 1.

**TABLE 1:** Participant demographic characteristics, transepidermal water loss rate, stratum corneum hydration, pH, erythema index, and CEA scores at baseline.

	Sensitive skin	Rosacea
Total	50	54
Gender, n		
Male	2	6
Female	48	48
Age, Mean (SD)	29.48 (8.44)	26.11 (5.41)
TEWL, Mean (SD)	31.21 (17.37)	29.05 (14.48)
SC hydration, Mean (SD)	54.13 (13.44)	56.66 (14.12)
PH, Mean (SD)	5.27 (0.72)	5.38 (1.12)
Erythema index, Mean (SD)	321.73 (76.34)	356.87 (73.13)
CEA scores,n (%)		
0 = clear	5 (10%)	1 (2%)
1 = almost clear	31 (62%)	24 (44%)
2 = mild	9 (18%)	26 (48%)
3 = moderate	5 (10%)	3 (6%)
4 = severe	0 (0%)	0 (0%)

Abbreviation: CEA, Clinician Erythema Assessment.

### 3.2 Biophysical Skin Measurements

**FIGURE 3:**
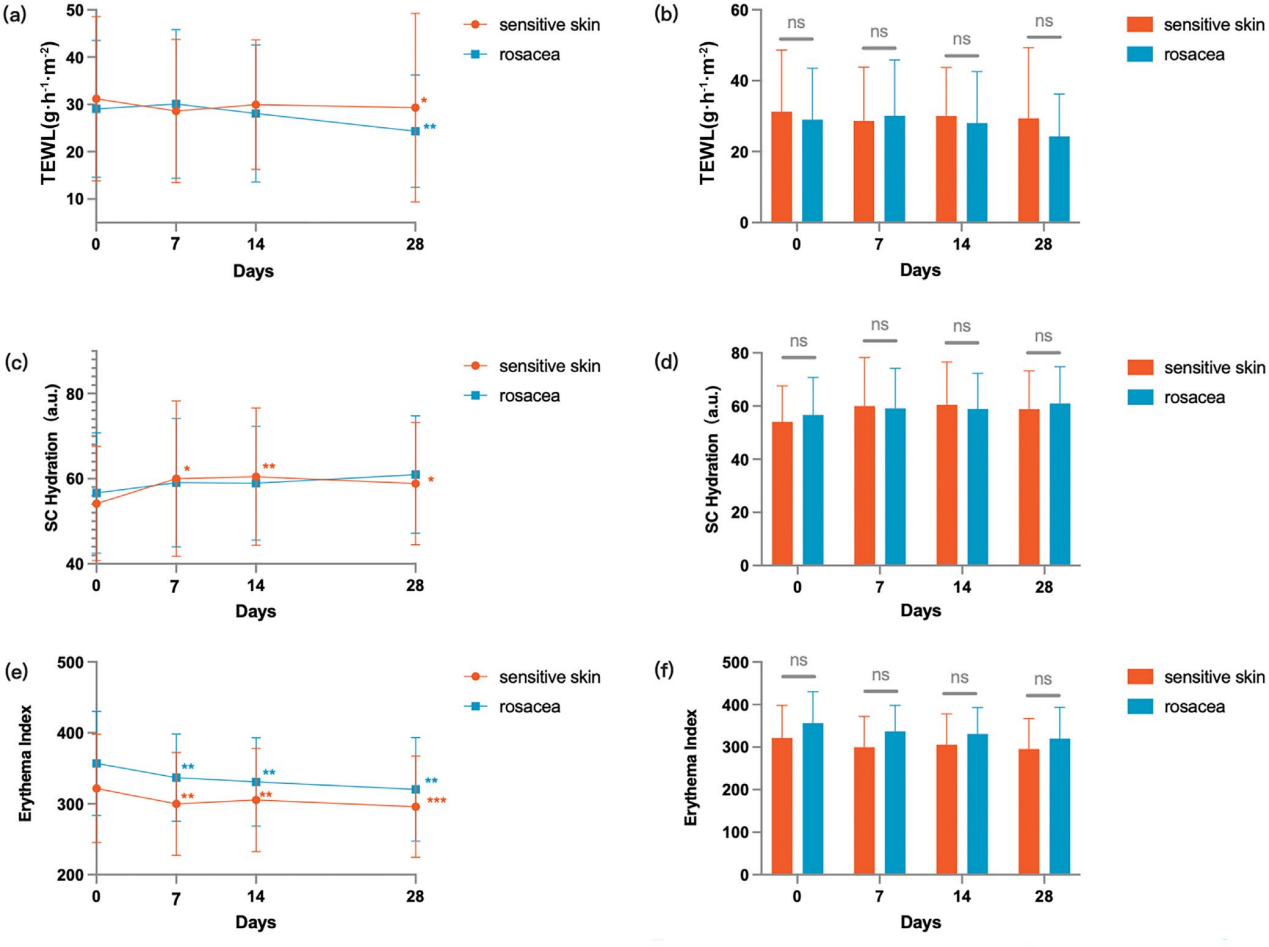
(a, c, e) Time‐course changes in transepidermal water loss (TEWL), stratum corneum (SC) hydration, and erythema index in the sensitive skin and rosacea groups. TEWL significantly decreased by Day 28 compared to baseline (D0) in both groups (*p* < 0.001). SC hydration significantly increased on Days 7, 14, and 28 in the sensitive skin group (*p* < 0.05, *p* < 0.01). Erythema index significantly decreased on Days 7, 14, and 28 in both groups (*p* < 0.01, *p* < 0.001). (b, d, f) Intergroup comparisons of TEWL, SC hydration, and erythema index at each time point. No significant differences were observed between the two groups (“ns”). *p < 0.05, **p < 0.01, ***p < 0.001.

The EI showed the most significant change among the physiological skin measurements. The EI of the two groups significantly decreased at 28 days compared with that at baseline (from 321.73 ± 76.34 to 295.80 ± 71.65 in sensitive skin and from 356.87 ± 73.13 to 320.30 ± 73.15 in rosacea); however, there were no significant differences between the groups (Figure 3, Table 2). The effect sizes indicated a small reduction for sensitive skin (Cohen's d = −0.35, 95% CI [−0.73, 0.03]) and a moderate reduction for rosacea (Cohen's d = −0.50, 95% CI [−0.90, −0.10]). Similarly, the TEWL of both groups significantly decreased on day 28 compared with that at baseline (*p* < 0.01 in rosacea; *p* < 0.05 in sensitive skin), but the difference between the groups was not significant (Cohen's d = −0.10, 95% CI [−0.48, 0.28] for sensitive skin; Cohen's d = −0.36, 95% CI [−0.75, 0.04] for rosacea). SC hydration increased on days 7, 14, and 28 in sensitive skin; these increases were significant (*p* < 0.05), with a small‐to‐moderate effect size at day 28 (Cohen's d = 0.34, 95% CI [−0.04, 0.72]). A slight increase in skin pH after product use was observed; however, all values were within the normal range (Cohen's d = 0.68, 95% CI [0.29, 1.08] for sensitive skin; Cohen's d = 0.47, 95% CI [0.06, 0.87] for rosacea).

**TABLE 2:** Changes in skin biophysical parameters over time in the sensitive skin group and the rosacea group. In the sensitive skin group, transepidermal water loss (TEWL) decreased significantly by Day 28 (*p* < 0.05). No significant changes were observed in the rosacea group (*p* > 0.05). Stratum Corneum (SC) hydration increased significantly in the sensitive skin group at Day 7 (*p* < 0.05), Day 14 (*p* < 0.01), and Day 28 (*p* < 0.05), while no significant changes were noted in the rosacea group (*p* > 0.05). Skin pH increased significantly in the sensitive skin group at Day 14 (*p* < 0.001), whereas in the rosacea group, pH increased significantly at Day 7, Day 14, and Day 28 (*p* < 0.01 for all). Sebum secretion showed no significant changes in the sensitive skin group (*p* > 0.05) but decreased significantly in the rosacea group at all timepoints (*p* < 0.05 or *p* < 0.01). The erythema index decreased significantly in the sensitive skin group across all timepoints, with the most significant reduction at Day 28 (*p* < 0.001). In the rosacea group, erythema significantly decreased only at Day 14 (*p* < 0.05) and Day 28 (*p* < 0.01).

		SENSITIVE SKIN				ROSACEA			
		Day 0	Day 7	Day 14	Day 28	Day 0	Day 7	Day 14	Day 28
TEWL	means ± SD	31.21 ± 17.37	28.62 ± 15.14	29.95 ± 13.71	29.33 ± 19.96	29.05 ± 14.48	30.11 ± 15.71	28.09 ± 14.49	24.34 ± 11.89
	Percentage of change	—	−8.30%	−4.04%	−6.02%	—	3.65%	−3.30%	−16.21%
	*p*	—	0.075	0.602	< 0.05*	—	0.82	0.337	< 0.01**
SC hydration	means ± SD	54.13 ± 13.44	60.01 ± 18.25	60.47 ± 16.12	58.85 ± 14.39	56.66 ± 14.42	59.07 ± 15.11	58.93 ± 13.40	60.99 ± 13.78
	Percentage of change	—	10.86%	11.71%	8.72%	—	4.25%	4.01%	7.64%
	*p*	—	< 0.05*	< 0.01**	< 0.05*	—	0.203	0.281	0.067
PH	means ± SD	5.27 ± 0.72	5.46 ± 0.65	5.80 ± 0.59	5.72 ± 0.59	5.38 ± 1.12	5.69 ± 0.91	5.77 ± 0.91	5.88 ± 1.03
	Percentage of change	—	3.61%	10.06%	8.54%	—	5.76%	7.25%	9.29%
	*p*	—	0.101	< 0.001***	< 0.001***	—	< 0.01**	< 0.01**	< 0.01**
Sebum secretion	means ± SD	78.04 ± 56.79	82.41 ± 74.52	85.04 ± 57.36	94.71 ± 69.35	94.47 ± 67.85	100.62 ± 64.94	107.37 ± 67.22	110.04 ± 71.90
	Percentage of change	—	5.60%	8.97%	21.36%	—	6.51%	13.66%	16.48%
	*p*	—	0.527	0.367	< 0.05*	—	0.982	0.238	0.410
Erythema index	means ± SD	321.73 ± 76.34	299.84 ± 72.63	305.46 ± 72.79	295.80 ± 71.65	356.87 ± 73.13	336.88 ± 61.40	330.97 ± 62.31	320.30 ± 73.15
	Percentage of change	—	−6.80%	−5.06%	−8.06%	—	−5.60%	−7.26%	−10.25%
	*p*	—	< 0.01**	< 0.01**	< 0.001***	—	< 0.01**	< 0.01**	< 0.01**

* *p* < 0.05.

** *p* < 0.01.

*** *p* < 0.001.

### 3.3 Erythema Assessment

**FIGURE 4:**
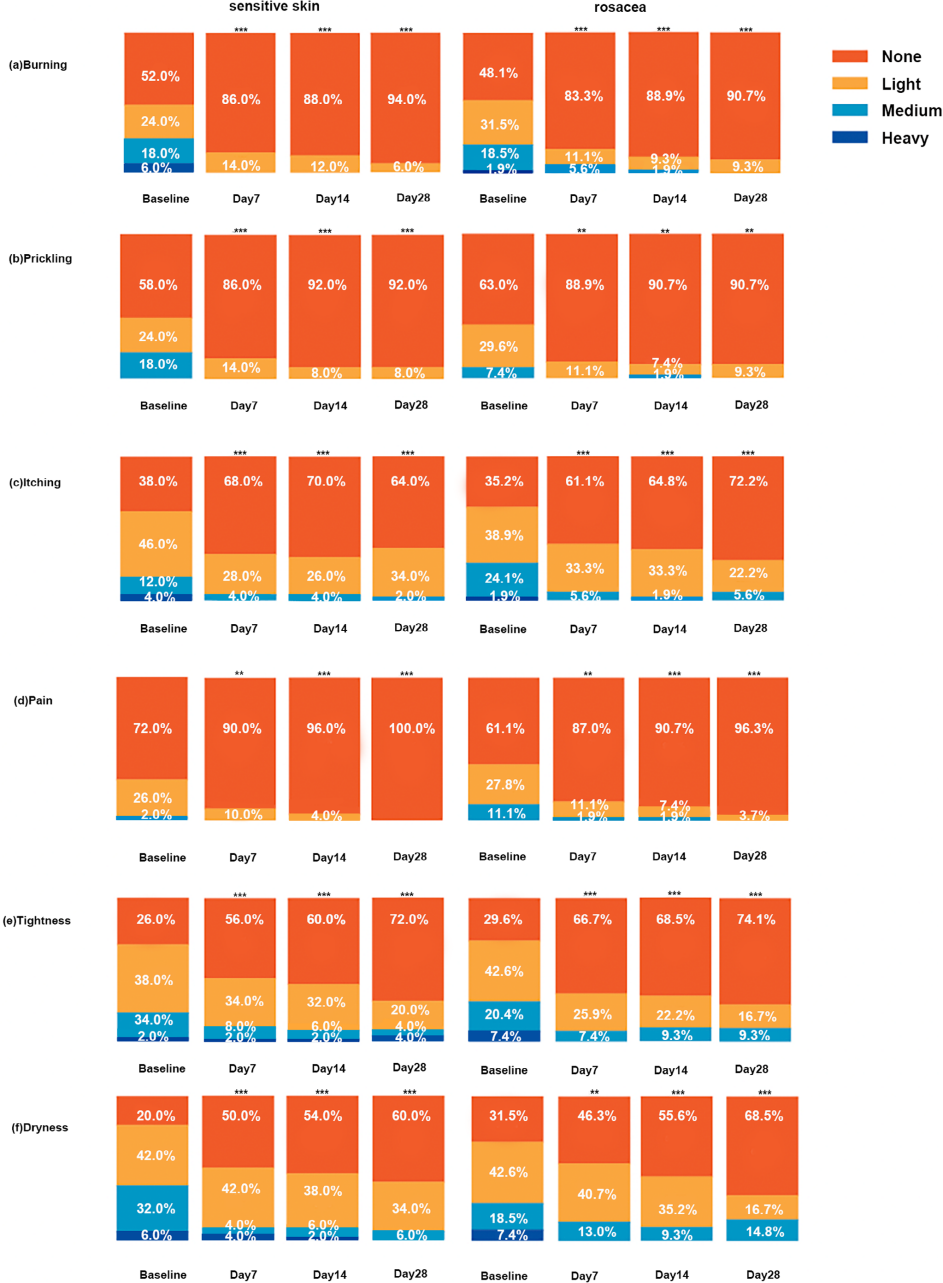
Percentage distribution of Clinician Erythema Assessment (CEA) scores at baseline (D0) and follow‐up visits (D7, D14, D28) in the sensitive skin and rosacea groups. CEA was rated on a 4‐point ordinal scale: 0 = clear (no visible erythema), 1 = almost clear (slight redness), 2 = mild (definite erythema), 3 = moderate (marked erythema). In the sensitive skin group, the proportion of lower scores (CEA 0–1) increased significantly at Days 7, 14, and 28 compared to baseline (*p* < 0.001). In the rosacea group, significant reductions in higher‐grade erythema (CEA 2–3) were observed at the same time points (*p* < 0.01). *p < 0.05, **p < 0.01, ***p < 0.001.

**FIGURE 5:**
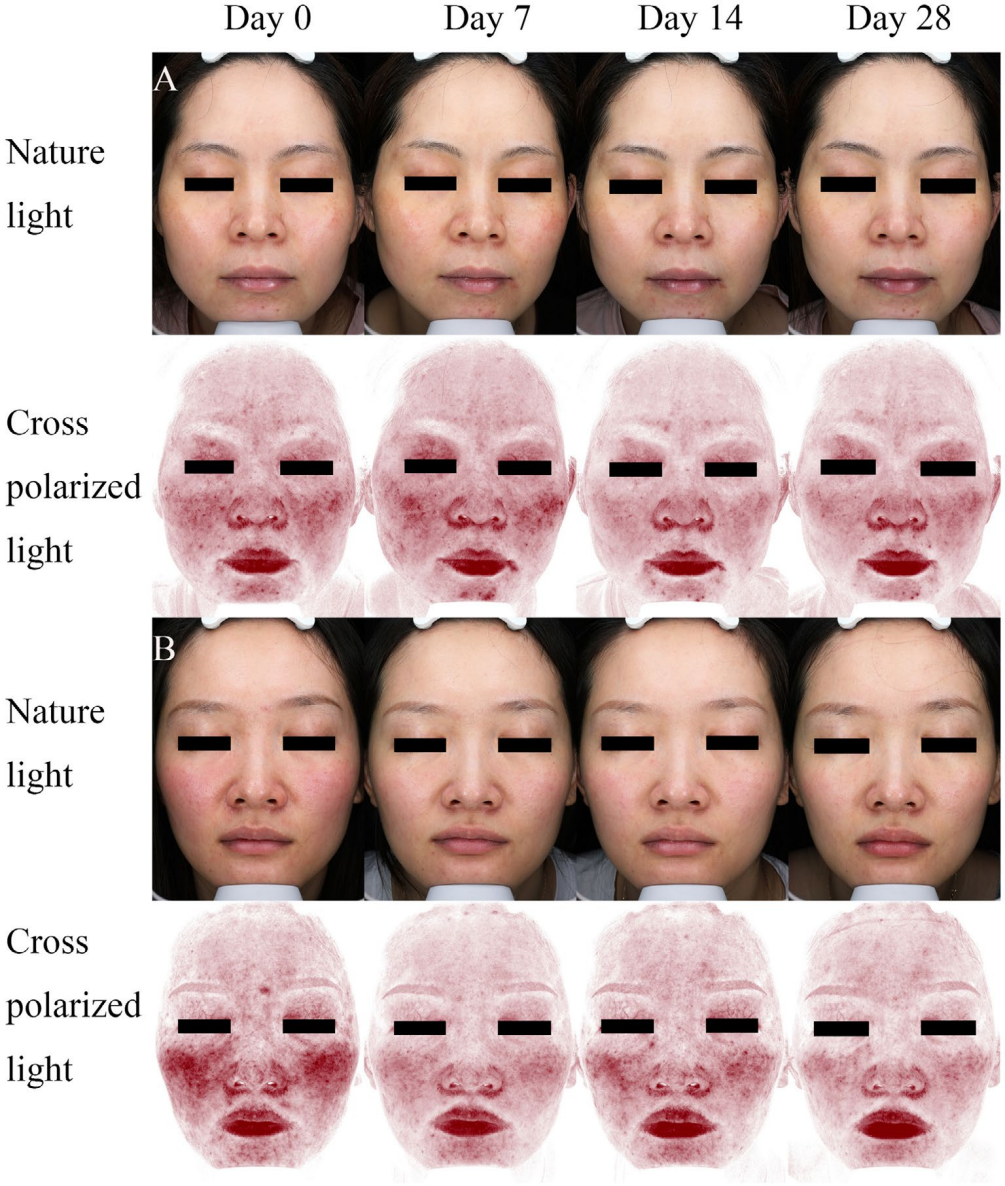
Typical images of both cheeks. Participant A has sensitive skin. Participant B has rosacea.

Objectively, the effect of treatment on erythema was confirmed by a decrease in the EI; subjectively, the treatment effect was verified by a decrease in the CEA score. On days 7, 14, and 28, the CEA scores in both groups were significantly lower than those at baseline (Figures 4, **5**) (*p* < 0.01).

### 3.4 Participant Self‐Assessment of Sensation

**FIGURE 6:**
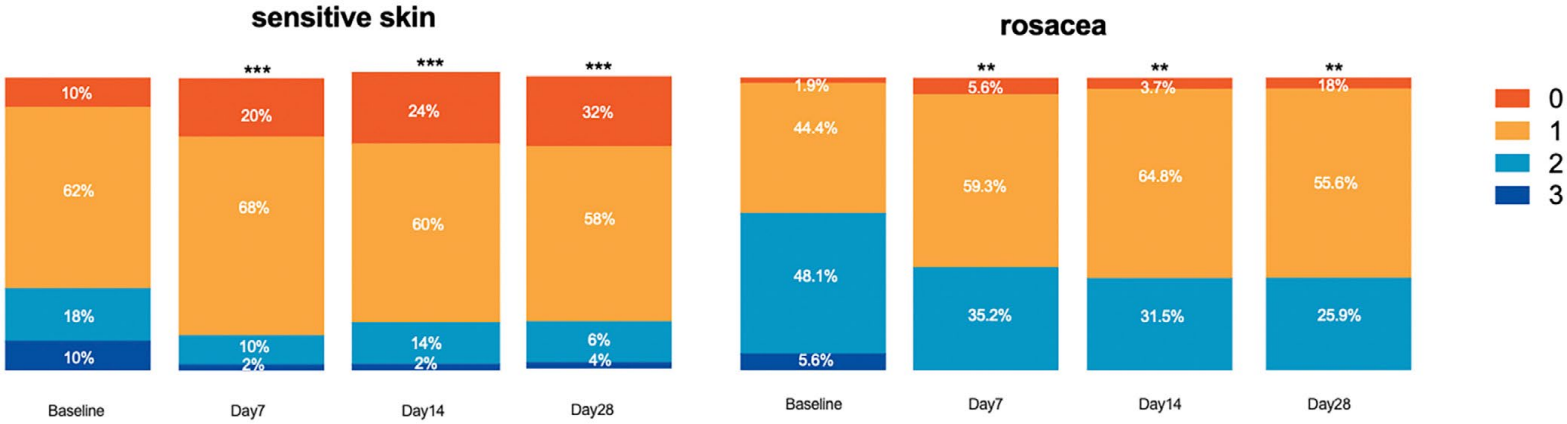
Self‐assessment of skin discomfort (burning, prickling, itching, pain, tightness, and dryness) at baseline (D0) and follow‐up visits (D7, D14, D28) in the sensitive skin and rosacea groups. Burning, itching, and tightness showed significant improvement in both groups at all follow‐up visits (*p* < 0.001). Prickling decreased significantly at all time points in the sensitive skin group (*p* < 0.001), and in the rosacea group (*p* < 0.01). Pain scores were significantly lower at Day 7 (*p* < 0.01) and further decreased at Days 14 and 28 (*p* < 0.001). Dryness improved significantly in the sensitive skin group at all time points (*p* < 0.001) and in the rosacea group at Day 7 (*p* < 0.01), with greater reductions at Days 14 and 28 (*p* < 0.001). *p < 0.05, **p < 0.01, ***p < 0.001.

The participants' sensations, including prickling, burning, itching, pain, self‐perceived dryness, and tightness, significantly improved (Figure 6, Table 3). However, there were no significant differences between the rosacea and sensitive skin groups.

**TABLE 3:** Changes in participant self‐assessment of sensation after intervention.

			SENSITIVE SKIN				ROSACEA			
			Day 0	Day 7	Day 14	Day 28	Day 0	Day 7	Day 14	Day 28
Burning	Graded	None	52.0%	86.0%	88.0%	94.0%	48.1%	83.3%	88.9%	90.7%
		Light	24.0%	14.0%	12.0%	6.0%	31.5%	11.1%	9.3%	9.3%
		Medium	18.0%	0.0%	0.0%	0.0%	18.5%	5.6%	1.9%	0.0%
		Heavy	6.0%	0.0%	0.0%	0.0%	1.9%	0.0%	0.0%	0.0%
	*p*		—	< 0.001***	< 0.001***	< 0.001***	—	< 0.001***	< 0.001***	< 0.001***
Prickling	Graded	None	58.0%	86.0%	92.0%	92.0%	63.0%	88.9%	90.7%	90.7%
		Light	24.0%	14.0%	8.0%	8.0%	29.6%	11.1%	7.4%	9.3%
		Medium	18.0%	0.0%	0.0%	0.0%	7.4%	0.0%	1.9%	0.0%
		Heavy	0.0%	0.0%	0.0%	0.0%	0.0%	0.0%	0.0%	0.0%
	*p*		—	< 0.001***	< 0.001***	< 0.001***	—	0.001**	0.004**	0.004**
Itching	Graded	None	38.0%	68.0%	70.0%	64.0%	35.2%	61.1%	64.8%	72.2%
		Light	46.0%	28.0%	26.0%	34.0%	38.9%	33.3%	33.3%	22.2%
		Medium	12.0%	4.0%	4.0%	2.0%	24.1%	5.6%	1.9%	5.6%
		Heavy	4.0%	0.0%	0.0%	0.0%	1.9%	0.0%	0.0%	0.0%
	*p*		—	< 0.001***	< 0.001***	< 0.001***	—	< 0.001***	< 0.001***	< 0.001***
Pain	Graded	None	72.0%	90.0%	96.0%	100.0%	61.1%	87.0%	90.7%	96.3%
		Light	26.0%	10.0%	4.0%	0.0%	27.8%	11.1%	7.4%	3.7%
		Medium	2.0%	0.0%	0.0%	0.0%	11.1%	1.9%	1.9%	0.0%
		Heavy	0.0%	0.0%	0.0%	0.0%	0.0%	0.0%	0.0%	0.0%
	*p*		—	0.009**	< 0.001***	< 0.001***	—	0.001**	< 0.001***	< 0.001***
Tightness	Graded	None	26.0%	56.0%	60.0%	72.0%	29.6%	66.7%	68.5%	74.1%
		Light	38.0%	34.0%	32.0%	20.0%	42.6%	25.9%	22.2%	16.7%
		Medium	34.0%	8.0%	6.0%	4.0%	20.4%	7.4%	9.3%	9.3%
		Heavy	2.0%	2.0%	2.0%	4.0%	7.4%	0.0%	0.0%	0.0%
	*p*		—	< 0.001***	< 0.001***	< 0.001***	—	< 0.001***	< 0.001***	< 0.001***
Dryness	Graded	None	20.0%	50.0%	54.0%	60.0%	31.5%	46.3%	55.6%	68.5%
		Light	42.0%	42.0%	38.0%	34.0%	42.6%	40.7%	35.2%	16.7%
		Medium	32.0%	4.0%	6.0%	6.0%	18.5%	13.0%	9.3%	14.8%
		Heavy	6.0%	4.0%	2.0%	0.0%	7.4%	0.0%	0.0%	0.0%
	*p*		—	< 0.001***	< 0.001***	< 0.001***	—	0.006**	< 0.001***	< 0.001***

*Note:* The table displays the percentage of participants reporting each level of sensation before (Baseline) and after (Follow‐up) the intervention. Data are presented as percentages of participants in each severity grade (none, light, medium, or heavy) for each symptom, including burning, prickling, itching, pain, tightness, and dryness, across all time points. *p* values indicate within‐group changes from baseline (Day 0). *p < 0.05; **p < 0.01; ***p < 0.001.

### 3.5 Dermatology Life Quality Index

In both groups, the mean total DLQI score at day 28 was significantly lower than that at baseline (*p* < 0.05), indicating an improvement in quality of life. In the sensitive skin group, the mean DLQI declined from 4.9 ± 4.4 at baseline to 2.3 ± 3.0 on day 28. In the rosacea group, the mean DLQI declined from 5.6 ± 4.1 at baseline to 3.1 ± 3.5 on day 28. Delta values were defined as the difference between the mean DLQI values at day 28 and baseline. No significant differences in delta values were observed between the groups (*p* > 0.05).

### 3.6 Safety Evaluation

No moderate or severe adverse events related to product use, such as erythema or tingling, were observed during the study period.

## 4 Discussion

The results of this study demonstrate that the novel composite herbal cream significantly improved clinical assessment scores (e.g., CEA), objective measurements (e.g., TEWL and EI), and subjective symptoms (stinging, burning, itching, dryness, and tightness) in individuals with sensitive skin and rosacea. These results suggest that simultaneously targeting skin barrier dysfunction and addressing clinical manifestations commonly associated with cutaneous inflammation offers an effective strategy for managing these intertwined skin conditions.

Rosacea pathophysiology involves a complex interplay of neurovascular dysregulation, altered innate immunity, and often a compromised epidermal barrier [22]. Similarly, although sensitive skin is commonly heterogeneous, impairment of epidermal barrier function serves as a central pathogenic mechanism [23]. Our findings align with this understanding, showing significant reductions in TEWL, which indicates barrier repair, along with decreased CEA scores, which reflect reduced clinical severity of erythema. The significant decrease in EI, observed as early as day 7 and sustained until days 14 and 28 in both groups, further supports this clinical observation. The marked alleviation of subjective sensory symptoms (e.g., stinging and burning), which are often linked to neurogenic inflammation, further implies beneficial effects of the novel herbal cream on the pathways involved in these skin conditions.

Several potential confounding factors must be considered when interpreting these findings. First, this was an open‐label, single‐arm study without a placebo control group. The absence of a placebo arm means that we cannot definitively exclude the possibility that the observed improvements were influenced by a placebo effect, the natural course of the conditions, or regression to the mean, particularly for subjective symptoms. The act of participating in a study and applying any cream, regardless of its active ingredients, can lead to perceived and even objective improvements. Second, the rosacea cohort was permitted to continue their standard medical treatments. Although this reflects a real‐world clinical scenario of adjunctive therapy, it makes it impossible to isolate the effect of the herbal cream from that of the concurrent medications. We demonstrated the apparent intrinsic efficacy of the cream in the sensitive‐skin group (who used it as a monotherapy). However, the magnitude of the synergistic or additive effect in the rosacea group remains unquantified. Future randomized, placebo‐controlled, and double‐blind studies are necessary to control for these variables and robustly validate the cream's efficacy. Furthermore, although the baseline characteristics between the two groups were generally similar, subtle differences in disease severity or lifestyle factors (e.g., diet, stress levels, and sun exposure) could have influenced the outcomes. We did not control for these environmental or behavioral factors, which are known to trigger flare‐ups in both sensitive skin and rosacea. These limitations underscore the preliminary nature of our findings and highlight the need for more rigorously controlled trials.

In comparison to commercially available barrier‐repair moisturizers marketed for rosacea, our novel cream shares a foundational strategy while offering a distinct mechanistic approach. Standard‐of‐care moisturizers for rosacea typically combine barrier‐replenishing lipids, such as ceramides, with specific anti‐inflammatory or neurosensory‐calming agents (e.g., licochalcone A) to manage erythema and sensitivity [24]. Our formulation aligns with this principle by incorporating exogenous ceramides [11]. However, it distinguishes itself through the integration of a unique combination of herbal actives. This blend of *Prinsepia utilis* oil, *Portulaca oleracea* extract, and *Physalis alkekengi* extract is designed to provide a multi‐targeted, synergistic effect—simultaneously enhancing endogenous barrier parameters, reducing inflammation, and mitigating erythema [10, 12, 13]. This contrasts with strategies that often rely on a single primary active ingredient to address inflammation. While direct head‐to‐head clinical comparisons are needed, recent reviews strongly support the rationale for such integrated approaches, re‐emphasizing the critical and complementary role of both barrier restoration (e.g., ceramides) and targeted anti‐inflammatory agents in rosacea management [25, 26].

The observed efficacy is attributed to the multi‐target actions of the herbal ingredients in the cream. For example, *P. oleracea* has demonstrated anti‐inflammatory functions [27, 28], and *P. alkekengi* constituents mediate anti‐inflammatory effects partly by inhibiting the NF‐κB pathway and downstream mediators such as TNF‐α [12, 13]. These documented anti‐inflammatory properties provide a plausible biological basis for the improved inflammation in the participants, which included erythema (CEA, EI) and related symptoms.

Beyond potential direct anti‐inflammatory effects, the improvement in TEWL highlights effective barrier restoration. *Prinsepia utilis* oil restores skin barrier function through coordinated regulation of corneocyte envelope proteins, lipid synthesis enzymes, and tight junction proteins [29]. Exogenous ceramides serve as precursors for endogenous ceramide synthesis and modulate keratinocyte differentiation to enhance epidermal barrier function [30]. Notably, barrier repair is not merely a secondary benefit; a restored barrier reduces TEWL, minimizes penetration by external irritants and microbes, and can itself modulate keratinocyte signaling to downregulate inflammation [31]. This bidirectional relationship between barrier function and inflammation may explain the synergistic effects observed in this study.

Comparing this herbal cream with standard therapies such as azelaic acid (AA) and metronidazole (MTZ) provides important context, as both are recommended first‐line treatments for reducing rosacea lesions and erythema [3]. However, unlike AA and MTZ, which do not primarily target barrier repair and may cause initial irritation, the herbal cream improved barrier function (demonstrated by significant TEWL reduction) while also exerting anti‐inflammatory effects. Direct statistical comparison with standard treatments was not feasible in the rosacea group due to concomitant therapies, but the significant improvements in the sensitive‐skin group using only the test cream—reductions in EI (−8.06%, *p* < 0.001) and TEWL (−6.02%, *p* < 0.05)—support its apparent intrinsic efficacy. The erythema reduction in the rosacea group (−10.25%, *p* < 0.01) should be interpreted in this context. Reported efficacy of AA and MTZ varies across studies, and definitive comparative conclusions about their relative efficacy would ideally rely on head‐to‐head randomized controlled trials [22]. The herbal cream demonstrated good tolerability (based on adverse event data). This favorable tolerability profile combined with the dual action of the cream (barrier repair plus improvement of symptoms associated with inflammation) suggests potential applications: (1) for patients intolerant to standard therapies or seeking gentler options; (2) as an adjunct to standard therapies to mitigate irritation and improve barrier function, potentially enhancing overall treatment adherence and outcomes, which is consistent with recommendations for comprehensive rosacea management [3]; and (3) for maintenance therapy focused on long‐term skin homeostasis and barrier health.

Previous research typically focused on either barrier repair primarily for sensitive skin or anti‐inflammatory agents primarily for rosacea flares. Our study is the first to include both patient groups and employ both clinical and objective assessments to track erythema, alongside barrier metrics and subjective symptoms. The likelihood of shared mechanisms underlying these skin conditions is reinforced by our findings that the cream effectively addressed both barrier issues (as shown by improved TEWL and reduced sensations of dryness and tightness) and clinical symptoms associated with inflammation (as shown by reduced CEA and EI scores and decreased stinging, burning, and itching) across both conditions, which implies the need for a holistic treatment approach. The lack of significant differences in efficacy between the sensitive‐skin and rosacea groups implies that the product targeted fundamental shared pathologies rather than disease‐specific triggers, warranting further investigation into potential differential effects between specific rosacea subtypes or sensitive‐skin phenotypes.

### 4.1 Limitations and Future Directions

This study provides promising preliminary data; however, several limitations must be acknowledged.

Its open‐label, single‐arm design without a placebo group means the cream's effects cannot be definitively separated from placebo responses or natural progression. In the rosacea group, concurrent medical therapies confound the results, making it impossible to isolate the cream's specific contribution. From a statistical perspective, two additional limitations should be noted. First, the sample size was not determined by a formal a priori power calculation but was based on precedent and feasibility considerations, which may limit power to detect smaller or more subtle effects. Second, given the exploratory nature of the analyses, no correction for multiple comparisons was applied; therefore, all reported *p*‐values are nominal, which may increase the risk of Type I error (false positives). Accordingly, these results should be interpreted cautiously as hypothesis‐generating rather than confirmatory. Other uncontrolled variables, including lifestyle and environmental/exposomal influences [32, 33], may also have influenced the outcomes. Lastly, direct mechanistic analyses were not conducted to confirm the suggested anti‐inflammatory pathways.

Future research should incorporate randomized, double‐blind, active‐controlled or placebo‐controlled designs with longer follow‐up periods to confirm our findings, assess long‐term efficacy and safety, including effects with continued maintenance use, and explicitly define the therapeutic benefits. Future studies should be designed with pre‐specified sample sizes based on formal power calculations to ensure adequate power to detect clinically meaningful differences. To control the risk of false positives due to multiple testing, confirmatory studies should apply appropriate correction methods, such as False Discovery Rate (FDR) correction. Incorporating direct mechanistic assessments would further elucidate the anti‐inflammatory and barrier‐repair mechanisms, and exploring the impact on the skin microbiome may provide additional insights. Additionally, stratifying participants according to rosacea subtype and sensitive skin subgroups in future trials could provide more targeted clinical insights.

## 5 Conclusion

The novel composite herbal cream is well‐tolerated and effective for improving the symptoms of both sensitive skin and rosacea. By concurrently enhancing skin barrier function and reducing clinical signs associated with inflammation, this product addresses key pathophysiological drivers of these skin conditions. The significant improvements observed even in the sensitive‐skin group underscore its inherent efficacy. Given its dual action and favorable tolerability, this herbal cream may serve as an adjunct or alternative therapy for patients with sensitive skin or rosacea, pending confirmation from future controlled trials.

## Author Contributions

Xiaohong Shu, Wei Huo, Zhaoxia Li, and Lin Zou performed the research; Li Li and Xi Wang designed the research; Yuling Chen, Qiujing Chen, and Wei Huo analyzed the data; Yuling Chen and Xi Wang wrote the manuscript; Xiaohong Shu, Li Li and Xi Wang reviewed and revised the manuscript. All authors have read and approved the final manuscript.

## Disclosure

Use of Artificial Intelligence Statement: DeepSeek was used solely for language editing, improvement of readability, and assistance with reference formatting during manuscript preparation. It was not used to generate scientific content, interpret data, draw conclusions, or select references. All AI‐assisted content and formatted references were carefully reviewed and verified by the authors, and any inaccuracies were corrected against the original sources to ensure the accuracy, validity, and integrity of the final manuscript.

## Ethics Statement

This study was conducted in accordance with the Declaration of Helsinki and was approved by the appropriate ethics review committee (Approval No. 2021审(360)号). Informed consent was obtained from all participants.

## Conflicts of Interest

The authors declare no conflicts of interest.

## Data Availability

The data that support the findings of this study are available from the corresponding author upon reasonable request.
